# Metabolic Syndrome Resolved within Two Years is Still a Risk Factor for Kidney Cancer

**DOI:** 10.3390/jcm8091329

**Published:** 2019-08-28

**Authors:** Tae Ryom Oh, Kyung-Do Han, Hong Sang Choi, Chang Seong Kim, Eun Hui Bae, Seong Kwon Ma, Soo Wan Kim

**Affiliations:** 1 Department of Internal Medicine, Chonnam National University Medical School, Gwangju 61469, Korea; 2 Department of Medical Statistics, College of Medicine, The Catholic University of Korea, Seoul 06591, Korea

**Keywords:** metabolic syndrome, kidney cancer, hypertension, abdominal obesity, improvement of metabolic syndrome

## Abstract

The prevalence of metabolic syndrome (MetS) and kidney cancer is increasing, but studies on the effects of MetS and its components on kidney cancer development have had ambiguous results. Overall, 7,613,865 patients from the Korean National Health Insurance System were analyzed and followed up until 2017. Patients with ≥3 of the necessary five components of MetS were diagnosed with MetS. Patients were divided into subgroups according to two consecutive physical examinations conducted every two years. The Cox proportional hazard regression model was used to survey the independent association between MetS and the risk of kidney cancer development. Kidney cancer risk was significantly higher in patients with MetS, and there was no difference according to sex. The hazards ratio of kidney cancer increased with increasing number of MetS components. For patients not diagnosed with MetS but with abdominal obesity and hypertension, the likelihood of developing kidney cancer was similar to that of patients diagnosed with MetS. Patients with improved MetS within two years had increased risk of kidney cancer compared with those without MetS. MetS is an independent risk factor for kidney cancer, and the obesity and hypertension components of MetS are also powerful risk factors.

## 1. Introduction

According to the GLOBOCAN report in 2018, kidney cancer is the 14th most common cancer with a global incidence of 403,262 (2.2%) [1]. In Korea, the incidence and prevalence of urological cancer have increased steadily over the past decade along with social changes such as westernized eating habits and increased life expectancy [2]. However, the etiology of kidney cancer is still unclear but several studies have shown that smoking and metabolic abnormalities such as obesity, hyperlipidemia, diabetes, and hypertension are associated with renal cell carcinoma (RCC) incidence [3,4,5,6].

Metabolic syndrome (MetS) is characterized by a combination of various metabolic abnormalities, including hyperglycemia, obesity, hypertension, glucose metabolism, and dyslipidemia. The prevalence of MetS in Korea is rapidly increasing, from 24.9% in 1998 to 31.3% in 2007 [7]. In some meta-analyses, a relationship between MetS and several cancers, including liver, colorectal, kidney, bladder, endometrial, postmenopausal breast, pancreatic, and colorectal cancer, has been observed [8,9,10,11]. In Koreans, the body mass index (BMI) was correlated with kidney cancer risk, but there was no correlation between fasting glucose and total cholesterol levels [12,13,14]. These studies have focused on the effect of only a single component, and there are only a few studies on the association between a combination of MetS components and kidney cancer. Additionally, MetS is a treatable disease; however, no studies have been conducted on its effects after improvement. Likewise, the risk of kidney cancer in patients recovering from MetS is unknown. The aim of this study was to identify the MetS components related to kidney cancer and to evaluate the relationship between MetS and kidney cancer, even if it improves within two years. To our knowledge, this is the first study to assess the association between the combination of MetS components and the risk of kidney cancer and the effect of recovery from MetS in an Asian population.

## 2. Material and Methods

### 2.1. Data Source and Study Population

We analyzed the database of the Korean National Health Insurance System (KNHIS), which covers almost all (approximately 97%) Korean citizens [15]. It is managed by KNHIS and has a wide range of data such as information on demographics, medical bills claimed by medical services, health examinations, and medical care institutions. Subscribers of the National Health Insurance Corporation are advised to undergo standardized medical examination at least every two years.

**Figure 1:**
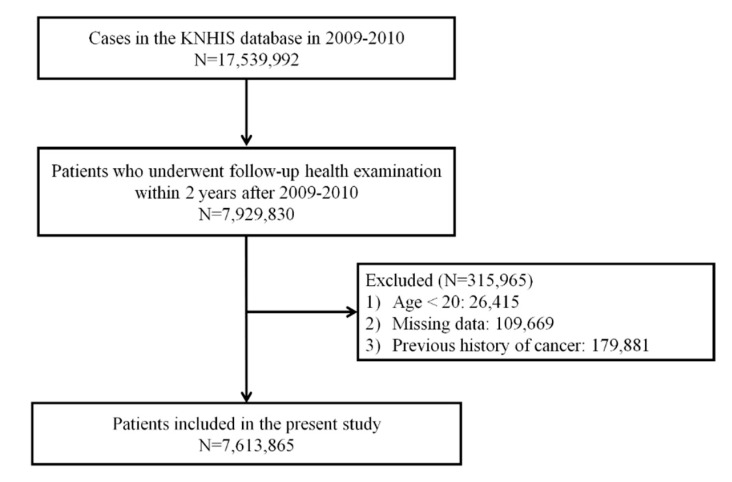
Flow diagram showing the study design.

Among 17,539,992 patients who underwent at least one health examination from 2009 to 2010 (index year), 9,610,162 cases who did not undergo follow-up health examination within two years ± 90 days after the health examination in the index year were excluded. Furthermore, 136,084 subjects with missing data or younger than 20 years and 179,881 subjects with history of cancer were excluded. A total of 7,613,865 patients were analyzed and followed up until 2017. Figure 1 shows the flowchart for selecting cases for this study.

### 2.2. End Point and Definitions

The primary end point of this study was newly diagnosed kidney cancer, which was defined using the combination of International Classification of Disease, 10th Revision (ICD-10) codes C64.1, C64.2, and C64.9. The diagnosis was considered new if the patient did not have such a diagnosis before 2009.

We used standardized self-reported questionnaires for age (years), sex, alcohol consumption (none; mild, <30 g of alcohol/day; heavy, ≥30 g of alcohol/day), and status of smoking (never, former, and current). Regular physical exercise was defined as regular strenuous exercise (high-intensity exericse ≥3 times/week or moderate-intensity exercise ≥5 times/week; none) [16]. We defined diabetes mellitus (DM) as at least one prescription of antidiabetic medication per year with ICD-10 codes E11-14 or fasting glucose level ≥126 mg/dL (from health examination data). Hypertension was defined as patients who had been prescribed antihypertensive agents with at least one claim per year with ICD-10 codes I10-15 or systolic/diastolic blood pressure of 140/90 mmHg [17].

We used the modified diagnostic criteria for MetS components recommended by the International Obesity Task Force of the Asia-Pacific region for Korean adults proposed by the Korean Society for the Study of Obesity [18], which include elevated blood pressure (systolic blood pressure ≥130 mmHg, diastolic blood pressure ≥85 mmHg) or history of treatment for hypertension, elevated triglyceride levels (≥150 mg/dL), decreased high-density lipoprotein (HDL) cholesterol levels (men, <40 mg/dL; women, <50 mg/dL), elevated fasting plasma glucose levels (≥100 mg/dL) or previously diagnosed type 2 DM, and abdominal obesity (waist circumference ≥90 cm for men and ≥85 cm for women). Patients with three or more of the five items were diagnosed with MetS.

Patients were divided into subgroups according to two consecutive health examinations conducted every two years. Patients who did not meet the diagnostic criteria for MetS in both consecutive screenings were assigned to the control group. Patients with MetS or its components in the index year and without MetS or its components in the next medical examination were assigned to the PRE group. Patients without MetS or its components in the index year and with MetS or its components in the next medical examination were assigned to the POST group. Patients with MetS or its components in two consecutive medical examinations were assigned to the BOTH group.

### 2.3. Statistical Analyses

Continuous variables were expressed as mean with standard deviation. Comparisons of continuous variables between two groups were performed using Student’s *t*-test. Categorical variables were described as the number of participants (percentage) and were compared using the chi-squared test between two groups. The incidence rates were calculated by dividing the number of events by the person–time at risk. Cox proportional hazard regression model was applied to survey the independent association between MetS and the risk of kidney cancer development. It was adjusted for age, sex, smoking status, alcohol consumption, BMI, and regular physical exercise, and the hazards ratio (HR) and 95% confidence interval (CI) were calculated. Interaction analysis was performed to observe the difference in the risk of kidney cancer according to sex. All statistical tests were two-tailed, and *p* < 0.05 was considered statistically significant. SAS version 9.3 software and SAS survey procedures (SAS Institute, Inc., Cary, NC, USA) were used for all statistical analyses.

### 2.4. Ethics Approval and Consent to Participate

This study adhered to the tenets of the Declaration of Helsinki. As the database used in this study did not include personal identifiers and the study was retrospective and observational in nature, the need for informed consent was waived. Ethical approval was given by the Chonnam National University Hospital Institutional Review Board (CNUHEXP-2018-276).

## 3. Results

### 3.1. Clinical Characteristics of the Participants

Of the 7,613,865 patients included, 2,212,857 (29.06%) were classified as having MetS based on the last medical examination. The baseline characteristics of the MetS and non-MetS groups are summarized in Supplementary Table S1. There were more women, ex/nonsmokers, heavy drinkers, and elderly people in the MetS group than in the non-MetS group. Higher prevalence of DM and hypertension, lower regular physical exercise and estimated glomerular filtration rate (eGFR), and greater waist circumference were observed in the MetS group than in the non-MetS group.

### 3.2. Association between Metabolic Syndrome and Kidney Cancer Development

Of the 2,212,857 patients with MetS, 3604 were newly diagnosed with kidney cancer. Meanwhile, of 5,401,008 subjects in the non-MetS group, 4060 patients were diagnosed with kidney cancer occurred. There was no significant difference in the mean follow-up period between the two groups (non-MetS group, 5.98 ± 0.66 years; MetS group, 5.94 ± 0.76 years).

**Figure 2:**
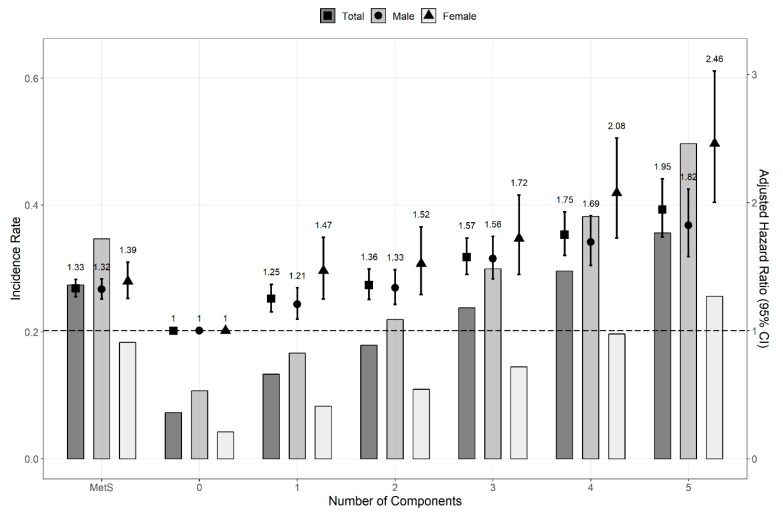
Hazard ratios and incidence rate for the risk of kidney cancer by numbers of MetS components (overall periods). Cox proportional hazard regression model was adjusted for age, sex, smoking status, alcohol consumption, body mass index, and regular physical exercise. When the analysis was conducted based on sex, the factor sex was excluded. Abbreviation: CI, confidence interval; MetS, metabolic syndrome.

**Figure 3:**
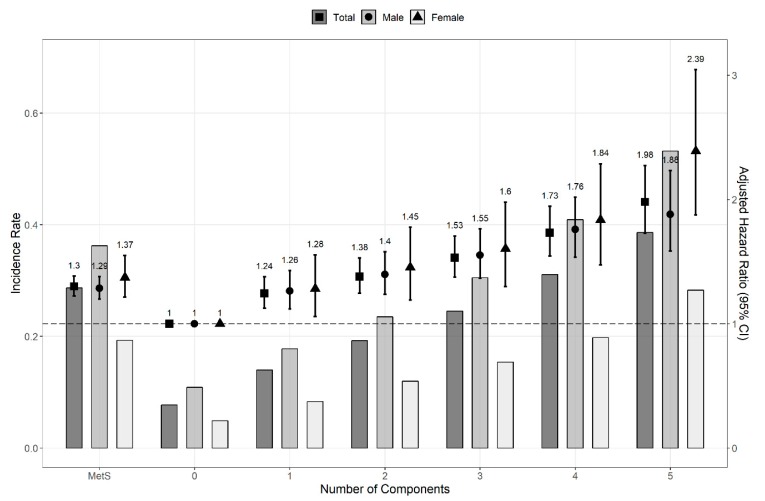
Hazard ratios and incidence rate for the risk of kidney cancer by numbers of MetS components (except for the first two years of follow-up). The Cox proportional hazard regression model was adjusted for age, sex, smoking status, alcohol consumption, body mass index, and regular physical exercise. When the analysis was conducted based on sex, the factor sex was excluded from the covariates. Abbreviation: CI, confidence interval; MetS, metabolic syndrome.

The HR and 95% CI were calculated with multivariate Cox proportional hazard regression models to compare the risk of kidney cancer. The risk of kidney cancer was significantly higher in patients with MetS (HR, 1.331; 95% CI, 1.265–1.400). We confirmed a consistent association between the risk of kidney cancer and MetS components in both sexes (men: HR, 1.322; CI, 1.245–1.402; women: HR, 1.386; 95% CI, 1.252–1.534; *p* for interaction, 0.009). The HRs of kidney cancer in both sexes increased as the number of MetS components increased (Figure 2). Regardless of whether the MetS diagnostic criteria were met or not, the HR (95% CI) of kidney cancer increased as the number of MetS components increased: 1.250 (1.147–1.362), 1.357 (1.244–1.480), 1.575 (1.439–1.724), 1.750 (1.588–1.927), and 1.946 (1.732–2.185) for 1, 2, 3, 4, and 5 components, respectively. Except for the first two years of follow-up, MetS components were also independent risk factors for kidney cancer throughout the overall period (Figure 3).

### 3.3. Risk of Kidney Cancer by Combinations of Metabolic Syndrome Components

Table 1 shows the incidence rate and adjusted multivariate HRs of the Cox proportional hazard models for the risk of kidney cancer using combinations of MetS components. Three MetS components (abdominal obesity, hypertension, and decreased HDL level) were related to an increased risk of development of kidney cancer. 

When the diagnostic criteria for MetS were not met, the combination of hypertension and abdominal obesity showed the strongest influence. The next strongest order of influence is the combination of hypertension and decreased HDL-cholesterol levels, hypertension and fasting glucose intolerance, and hypertension and hypertriglyceridemia.

When the MetS diagnostic criteria were met, the combination of hypertension, fasting glucose intolerance, and decreased HDL-cholesterol levels showed the greatest impact on the risk of kidney cancer development. Except for the two combinations—(1) abdominal obesity, fasting glucose intolerance, and hypertriglyceridemia and (2) abdominal obesity, fasting glucose intolerance, and decreased HDL-cholesterol level—all other combinations were statistically significant.

### 3.4. Subgroup Analysis by Recovery from Metabolic Syndrome

**Figure 4:**
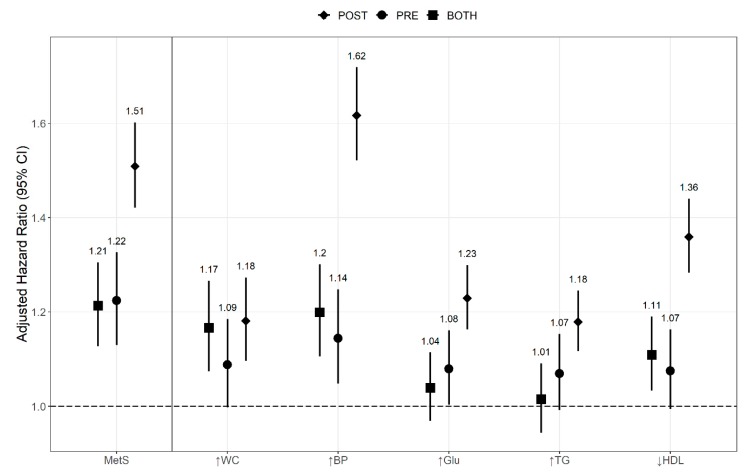
Hazard ratios for the risk of kidney cancer by recovery from MetS and its components. The Cox proportional hazard regression model was adjusted for age, sex, smoking status, alcohol consumption, body mass index, and regular physical exercise. Abbreviation: CI, confidence interval; ↑WC, abdominal obesity; ↑BP, hypertension; ↑Glu, impaired glucose tolerance; ↑TG, hypertriglyceridemia; ↓HDL, decreased HDL-cholesterol; MetS, metabolic syndrome.

When compared to the control group, the POST, PRE, and BOTH groups showed poor outcome in patients with MetS, with HR (95% CI) of 1.213 (1.127–1.305), 1.224 (1.130–1.325), and 1.509 (1.421–1.602), respectively (Figure 4). In each component of MetS, as expected, the BOTH group showed the worst results. The PRE group showed that hypertension and fasting glucose intolerance were related with kidney cancer, with HR (95% CI) of 1.144 (1.048–1.248) and 1.179 (1.117–1.245), respectively. The POST group showed that hypertension, abdominal obesity, and decreased HDL-cholesterol level were associated with kidney cancer, with HR (95% CI) of 1.199 (1.106–1.301), 1.166 (1.074–1.266), and 1.109 (1.033–1.190), respectively. Interestingly, even if MetS had improved within two years (PRE group), the risk of kidney cancer increased, compared with those without MetS (control group).

## 4. Discussion

In this nationwide study, MetS was closely related with the risk of kidney cancer, and there were no significant differences in the influence of MetS on either sex-specific development of kidney cancer. When the MetS diagnostic criteria were met (≥3 out of 5 components), the combination of abdominal obesity, hypertension, and decreased HDL-cholesterol levels showed the strongest association. Compared with the case without MetS, we observed that the risk of kidney cancer increased despite the improvement of MetS.

Several studies have evaluated the association between kidney cancer risk and MetS components. Most cancers that develop in the kidneys are RCCs; thus, most of the studies are limited to RCC. From a Swedish cohort, it was reported that higher BMI and hypertension are significant risk factors of RCC for men [19]. A meta-analysis of 22 clinical studies available in MEDLINE from 1966 to 1998 showed that increased BMI is equally associated with increased risk of RCC in both sexes [20]. The findings from studies for Caucasians, African-Americans, and Chinese showed a strong relationship between increased blood pressure and higher risk of RCC [21,22]. A total of 153,852 Swedish people were analyzed in a study of the general population, and results showed that the morbidity and mortality of RCC increased in diabetic patients [23]. Furthermore, Joh et al. [24] reported that type 2 DM was a risk factor for RCC in women. However, in terms of dyslipidemia, only a few studies have been conducted and the results are inconsistent [25,26,27]. Because most of the above studies did not include an Asian population, the consideration of racial differences is insufficient. Additionally, they did not survey the combination effect of the MetS components.

Recently, the plausibility of a biomolecular basis for the association between MetS and kidney cancer was studied [28,29,30,31,32]. Hyperinsulinemia and insulin resistance are closely related to the development of MetS. The insulin-like growth factor family, which is affected by insulin resistance, may play an important role in cellular mitosis, migration, and inhibition of apoptosis through mitogen-activated protein kinase and phosphatidylinositol 3 kinase pathways [28]. Obesity can lead to tissue hypoxia, followed by induction of a series of inflammatory cytokines, such as tumor necrosis factor-α (TNF-α) and interleukins [29,30]. In addition, TNF-α may induce the epithelial-mesenchymal transition of RCC with the help of glycogen synthase kinase 3β, suggesting its participation in RCC proliferation and metastasis [31]. Increased IL-6 is known to be associated with invasiveness, metastasis, and prognosis of renal cell carcinoma. [32]. It is well known that COX-2 is highly expressed in adipocytes and associated with RCC and insulin resistance [33,34,35]. Increased COX-2 and TNF-α mRNA expression in adipocytes in high-fat rats have been reported [36]. These changes inhibit lipogenesis, adipogenesis, and lipolysis [37]. The use of COX-2 inhibitors in this situation has been reported to inhibit the response in animal experiments. Based on these results, it can be inferred that there is COX-2 activation of visceral fat induced insulin resistance through the generation of systemic inflammatory TNF-α [36]. Furthermore, adiponectin, which is decreased in obese patients, can inhibit in vitro tumor growth through an adenosine monophosphate (AMP)-activated protein kinase and act as an inhibitor of cancer angiogenesis [38,39]. Peroxisome proliferator-activated receptors (PPARs) consist of three subtypes: PPARα, PPARβ, and PPARγ. Among them, PPARγ has been shown to ameliorate insulin resistance and regulate adipocyte differentiation [40,41,42]. The expression of PPAR increases in RCC tissues, and PPAR induces cellular apoptosis and inhibits the proliferation of RCC [43,44]. In experiments with cultured adipocytes, HIF-1α expression was upregulated by hypoxia [45]. Alternation of HIF-1 signaling resulted in a decrease in insulin secretion from the pancreatic β cells and insulin resistance through adipocyte dysfunction [46]. In animal studies, increased HIF levels have been shown to promote expression of the aryl hydrocarbon receptor (AhR) nuclear translocator (ARNT) and enhance the function of pancreatic β cells [47,48]. HIF-1α has been shown not only to increase intravascular tumor microvascular density in xenografts but also to overexpress in RCC [49]. These biological links may give us a clue to the causality between kidney cancer and MetS.

Patients with hypertension and abdominal obesity who did not meet the diagnostic criteria of MetS had the same risk of developing kidney cancer as patients with MetS (Table 1). Notably, regardless of sex, the presence of only one MetS component can significantly increase the HR of kidney cancer (Figure 2). The HR by number of MetS components is higher in women than in men, and the incidence in women is relatively lower than that in men.

Although MetS may have improved within two years, it has an impact as a risk factor for kidney cancer (Figure 4). In the case of recovery from MetS (PRE group), the HR remained higher than that of the control group, and there was no significant difference when compared with the POST group. It is an interesting result, indicating that even if there is a recovery from MetS, it was still a risk factor for kidney cancer. This phenomenon may be related to metabolic memory. It refers to the concept that when hyperglycemia occurs, a series of intracellular protein reactions that occur remain as memories and affect long-term complications. As a follow-up to the Diabetes Complications and Control Trial (DCCT), the Epidemiology of Diabetes Interventions and Complications (EDIC) trial showed that patients who received standard therapy during the DCCT and subsequently switched to intensive therapy had a higher incidence of diabetic complications than did the patients receiving intensive therapy throughout the trial [50,51]. These clinical trials on diabetes suggested a metabolic memory. In recent studies, excess superoxide anion in the mitochondria of endothelial cells produced in response to hyperglycemia has been reported to affect the development of diabetic complications [52]. A potential mechanism to time discrepancy between the hypothesis of superoxide excess and the metabolic memory phenomenon is that nucleic acids, proteins, and lipid proteins that are targets of superoxide and reactive species have a long half-life [53]. In addition, chronic hyperglycemia is thought to alter mitochondrial function through glucose modification of mitochondrial proteins. Glycated proteins or lipids are called advanced glycation end-products and are known to play a causative role in diabetic complications [54]. The formation of mitochondrial advanced glycation end-products are considered an irreversible process and could be the reason for the long-term nature of the metabolic memory [53]. Further, epigenetic mechanisms have been recognized as important interfaces between genetic and environmental factors in order to explain metabolic memory [55,56,57]. Hyperglycemia can irreversibly change the activity of post-translational histone modifications and DNA methyltransferases, and these alternations may explain the long-term harmful effects of metabolic memory [58,59,60].

Based on the results of this study and metabolic memory, we can infer that more careful observation is needed for patients with a history of MetS relative to the general population, and further studies are needed. Although the Korean National Health Insurance System database has many strengths, including a large-scale, nationwide observational design, robust data collection, and validated follow-up duration (approximately six years), our analyses have some limitations. First, as in all observation studies, we could not assess the precise causality between MetS and development of kidney cancer. However, observational studies are powerful tools in assessing epidemiologic relationships, and we capitalized on complimentary analytic methods to robustly examine the relationship between MetS and kidney cancer [61]. Second, this study could not solve all the problems of hidden bias and confounding factors. Socioeconomic status and smoking intensity are well known to be closely related with cancer development, and adjustment for these factors was insufficient due to data limitation in this study. Third, because our study was a retrospective one using a registry, there was a possibility that bias had occurred due to overdiagnosis/underdiagnosis or misclassification of patients. Fourth, our study lacked data on the histological type or stage of kidney cancer. Therefore, although the subtype and stage of cancer were important, the analysis of the effects of MetS on each subtype and stage of kidney cancer was limited. Recent studies have reported that the obesity paradox has different effects depending on the histologic type or stage of cancer [62]; therefore, further research on the obesity paradox is needed.

## 5. Conclusions

In summary, MetS is an independent risk factor for kidney cancer, and its obesity and hypertension components are also important risk factors. Due to the maintained effect of MetS, patients with a history of MetS may require more stringent screening tests for cancer than the general population, and this should be considered in policy making related to cancer screening. Considering the link between the molecular biology of MetS and kidney cancer, further development of targeted therapeutic intervention may inhibit carcinogenesis; hence, further studies are needed in this direction.

## Figures and Tables

**Table 1:** Multivariate adjusted Cox proportional hazard regression models and incidence rates according to the combination of MetS components.

Combination of Components					No. of Patients	Events	Duration (Person-Year)	IR	HR (95% CI)	
↑WC	↑BP	↑Glu	↑TG	↓HDL					Model 1	Model 2
**-**	**-**	**-**	**-**	**-**	2,004,634	882	11,999,208	0.1	1 (ref.)	1 (ref.)
**+**	**-**	**-**	**-**	**-**	149,029	134	891,292	0.2	1.626 (1.356, 1.95)	1.305 (1.083, 1.573)
**-**	**+**	**-**	**-**	**-**	773,460	798	4,634,552	0.2	1.477 (1.34, 1.628)	1.401 (1.271, 1.545)
**-**	**-**	**+**	**-**	**-**	425,976	297	2,541,016	0.1	1.139 (0.998, 1.3)	1.103 (0.966, 1.258)
**-**	**-**	**-**	**+**	**-**	325,136	189	1,966,854	0.1	1.017 (0.869, 1.19)	0.951 (0.812, 1.114)
**-**	**-**	**-**	**-**	**+**	281,701	147	1,677,871	0.1	1.306 (1.096, 1.556)	1.26 (1.057, 1.501)
**+**	**+**	**-**	**-**	**-**	170,219	300	1,015,406	0.3	2.339 (2.049, 2.671)	1.82 (1.58, 2.096)
**+**	**-**	**+**	**-**	**-**	61,976	63	367,964	0.2	1.47 (1.138, 1.899)	1.164 (0.898, 1.51)
**+**	**-**	**-**	**+**	**-**	74657	61	449,502	0.1	1.419 (1.094, 1.839)	1.11 (0.852, 1.445)
**+**	**-**	**-**	**-**	**+**	38,079	28	226,804	0.1	1.465 (1.006, 2.135)	1.155 (0.791, 1.688)
**-**	**+**	**+**	**-**	**-**	384,905	518	2,283,741	0.2	1.545 (1.382, 1.727)	1.443 (1.29, 1.614)
**-**	**+**	**-**	**+**	**-**	256,457	259	1,548,799	0.2	1.404 (1.221, 1.614)	1.275 (1.108, 1.468)
**-**	**+**	**-**	**-**	**+**	119,524	147	710,634	0.2	1.9 (1.593, 2.266)	1.74 (1.458, 2.077)
**-**	**-**	**+**	**+**	**-**	142,295	105	853,799	0.1	1.091 (0.891, 1.336)	1.002 (0.818, 1.228)
**-**	**-**	**+**	**-**	**+**	63,570	47	376,141	0.1	1.38 (1.029, 1.851)	1.291 (0.962, 1.732)
**-**	**-**	**-**	**+**	**+**	260,763	177	1,563,409	0.1	1.165 (0.991, 1.369)	1.076 (0.915, 1.266)
**+**	**+**	**+**	**-**	**-**	134,501	260	794,356	0.3	2.143 (1.861, 2.467)	1.65 (1.421, 1.917)
**+**	**+**	**-**	**+**	**-**	112,618	161	676,676	0.2	2.01 (1.698, 2.379)	1.531 (1.283, 1.827)
**+**	**+**	**-**	**-**	**+**	44,562	83	265,050	0.3	2.661 (2.122, 3.338)	2.034 (1.612, 2.565)
**+**	**-**	**+**	**+**	**-**	47,450	54	283,290	0.2	1.662 (1.262, 2.188)	1.288 (0.974, 1.703)
**+**	**-**	**+**	**-**	**+**	16,061	17	94,825	0.2	1.733 (1.072, 2.801)	1.343 (0.829, 2.177)
**+**	**-**	**-**	**+**	**+**	67,816	78	406,884	0.2	1.856 (1.472, 2.34)	1.443 (1.139, 1.828)
**-**	**+**	**+**	**+**	**-**	203,258	302	1,213,205	0.2	1.775 (1.555, 2.026)	1.597 (1.397, 1.826)
**-**	**+**	**+**	**-**	**+**	63,859	123	374,632	0.3	2.435 (2.012, 2.947)	2.204 (1.82, 2.67)
**-**	**+**	**-**	**+**	**+**	311,316	456	1,857,688	0.2	1.886 (1.68, 2.117)	1.692 (1.505, 1.902)
**-**	**-**	**+**	**+**	**+**	142,669	153	849,343	0.2	1.54 (1.296, 1.83)	1.404 (1.181, 1.67)
**+**	**+**	**+**	**+**	**-**	122,496	234	727,740	0.3	2.306 (1.993, 2.667)	1.749 (1.497, 2.043)
**+**	**+**	**+**	**-**	**+**	37,517	68	220,440	0.3	2.22 (1.731, 2.846)	1.675 (1.299, 2.161)
**+**	**+**	**-**	**+**	**+**	164,708	302	982,987	0.3	2.32 (2.031, 2.649)	1.756 (1.522, 2.026)
**+**	**-**	**+**	**+**	**+**	56,178	72	334,718	0.2	1.785 (1.403, 2.272)	1.377 (1.077, 1.761)
**-**	**+**	**+**	**+**	**+**	313,617	588	1,853,865	0.3	2.129 (1.912, 2.371)	1.896 (1.699, 2.115)
**+**	**+**	**+**	**+**	**+**	242,858	561	1,435,002	0.4	2.656 (2.381, 2.962)	1.991 (1.761, 2.25)

Model 1 is adjusted with age and sex. Model 2 is adjusted with age, sex, smoking status, alcohol consumption, and regular physical exercise and body mass index. Abbreviation: IR, incidence rate; HR, hazard ratio; CI, confidence interval; ref, reference; ↑WC, abdominal obesity; ↑BP, hypertension; ↑Glu, impaired glucose tolerance; ↑TG, hypertriglyceridemia; ↓HDL, decreased HDL-cholesterol; MetS, metabolic syndrome.

## Supplementary Materials

The following are available online at https://www.mdpi.com/2077-0383/8/9/1329/s1.
