# Supplementary material for: Metabolic Syndrome Resolved within Two Years is Still a Risk Factor for Kidney Cancer
Source: J Clin Med. 2019 Aug 28;8(9):1329. doi: 10.3390/jcm8091329 (PMC6780562; doi:10.3390/jcm8091329)
Supplement: Supplementary file 1 [file jcm-08-01329-s001.pdf]

Supplementary Table 1. Clinical characteristics of study population.

	non-MetS	MetS	*p*-Value
	(*n* = 5,401,008)	(*n* = 2,212,857)	
Age	46.23 ± 13.05	56.17 ± 12.84	< 0.001
Age > 60 years old (%)	776,162 (14.37)	842,708 (38.08)	< 0.001
Male (%)	3,030,285 (56.11)	1,227,539 (55.47)	< 0.001
Height (cm)	164.71 ± 8.96	162.71 ± 9.85	< 0.001
Weight (cm)	62.74 ± 10.93	68.62 ± 12.56	< 0.001
Waist circumference (cm)	78.12 ± 8.2	86.62 ± 8.07	< 0.001
Body mass index (%)	23.02 ± 2.84	25.77 ± 3.11	< 0.001
Diabetes mellitus (%)	171,270 (3.17)	543,822 (24.58)	< 0.001
Hypertension (%)	776,695 (14.38)	1,296,703 (58.6)	< 0.001
Systolic blood pressure (mmHg)	119.13 ± 13.52	130.48 ± 14.17	< 0.001
Diastolic blood pressure (mmHg)	74.59 ± 9.31	80.49 ± 9.76	< 0.001
eGFR (mL/min/1.73 m^2)			
<60	192,963 (3.57)	190,573 (8.61)	< 0.001
60–90	2,703,995 (50.06)	1,224,172 (55.32)	
≥90	2,504,050 (46.36)	798,112 (36.07)	
Mean	91.1 ± 36.65	85.87±35.05	< 0.001
Smoker (%)			< 0.001
Non	3,222,082 (59.66)	1,301,596 (58.82)	
Ex	865,614 (16.03)	414,262 (18.72)	
Current	1,313,312 (24.32)	496,999 (22.46)	
Drinker (%)			< 0.001
Non	2,633,594 (48.76)	1,244,913 (56.26)	
Mild	2,460,598 (45.56)	808,099 (36.52)	
Heavy	306,816 (5.68)	159,845 (7.22)	
Dyslipidemia (%)	533,416 (9.88)	1,082,094 (48.92)	< 0.001
Regular physical exercise (%)	3,110,696 (57.59)	1,149,483 (51.95)	< 0.001
Follow-up duration (year)	5.98 ± 0.66	5.94±0.76	< 0.001

Comparison between the two groups was performed using Student's *t*-test for continuous variables and chi-squared test for categorical variables. Abbreviation: MetS, metabolic syndrome; eGFR, estimated glomerular filtration rate.
